# Rapid Characterization of Biomolecules’ Thermal Stability in a Segmented Flow-Through Optofluidic Microsystem

**DOI:** 10.1038/s41598-020-63620-5

**Published:** 2020-04-24

**Authors:** Zdenka Fohlerova, Hanliang Zhu, Jaromir Hubalek, Sheng Ni, Levent Yobas, Pavel Podesva, Alexandr Otahal, Pavel Neuzil

**Affiliations:** 1 Central European Institute of Technology, Brno University of Technology, Purkynova 123, 612 00 Brno, Czech Republic; 2 Department of Microelectronics, Faculty of Electrical Engineering and Communication, Brno University of Technology, Technicka 3058/10, 61600 Brno, Czech Republic; 3 Ministry of Education Key Laboratory of Micro and Nano Systems for Aerospace, School of Mechanical Engineering, Northwestern Polytechnical University, 127 West Youyi Road, Xi’an, Shaanxi 710072 P.R. China; 4 Hong Kong, University of Science and Technology, Clear Water Bay, Hong Kong, P.R. China

**Keywords:** Biochemistry, Biological techniques, Nanoscience and technology

## Abstract

Optofluidic devices combining optics and microfluidics have recently attracted attention for biomolecular analysis due to their high detection sensitivity. Here, we show a silicon chip with tubular microchannels buried inside the substrate featuring temperature gradient (∇*T*) along the microchannel. We set up an optical fluorescence system consisting of a power-modulated laser light source of 470 nm coupled to the microchannel serving as a light guide via optical fiber. Fluorescence was detected on the other side of the microchannel using a photomultiplier tube connected to an optical fiber via a fluorescein isothiocyanate filter. The PMT output was connected to a lock-in amplifier for signal processing. We performed a melting curve analysis of a short dsDNA – SYBR Green I complex with a known melting temperature (*T*_M_) in a flow-through configuration without gradient to verify the functionality of the proposed detection system. We then used the segmented flow configuration and measured the fluorescence amplitude of a droplet exposed to ∇*T* of ≈ 2.31 °C mm^−1^, determining the heat transfer time as ≈ 554 ms. The proposed platform can be used as a fast and cost-effective system for performing either MCA of dsDNAs or for measuring protein unfolding for drug-screening applications.

## Introduction

Temperature can significantly affect the biological systems of living organisms in terms of cellular morphology, metabolism, growth, and cell death^1,2^. On a molecular level, temperature influences the structure and function of biomolecules such as proteins and nucleic acid^3,4^. The denaturation of biomolecules under relatively high temperatures is utilized in real-time polymerase reactions (qPCR), subsequent melting curve analyses (MCA) of double-stranded deoxyribonucleic acid (dsDNA), and differential scanning fluorimetry (DSF) of proteins.

The MCA analysis of dsDNA stained with an intercalator dye such as SYBR Green I is conducted by gradually ramping the temperature while monitoring the intensity of the emitted fluorescence (*F*). The dsDNA melting temperature (*T*_M_) is defined as the temperature at which 50% of presented dsDNA molecules separate into single-stranded forms. This value depends on the number of base pairs and their composition. Typically, while the MCA is performed using the qPCR systems, they can be also used to determine the protein-unfolding curve. However, qPCR systems currently available are bulky, costly, and slow.

Advances in microfabrication technology have enabled the miniaturization of bioanalytical tools with precisely controlled temperatures, allowing for quick investigations into the thermal effects on biological molecules using samples with a small volume. Heating methods and the thermal conductivity of the device’s materials are essential to create thermally stable systems^5^ with either homogeneous distribution of temperature or temperature gradient (∇*T*).

Heat conduction-based microfluidic techniques, especially in a droplet configuration, have attracted attention due to their applicability in DNA amplification, protein analysis, single-cell assays, and chemical synthesis. The characteristics of the thermal behavior of water in oil droplets within microfluidic channels were studied per the generation and transport of liquid droplets within local heating at the breakup location of T-junctions and flow-focusing systems^6,7^ either at the downstream channel^8,9^ or with the heater placed at the end of the transport channel^10^. However, the temperature dependency of droplet physical properties represented by density, viscosity, and interfacial tension between the continuous oil phase and water-based droplet may complicate high-performance droplet microfluidics. Further, the mathematical simulation of droplet behavior under heating is complicated due to the necessity of 3D models of a droplet-based system^11^. In addition to the investigation of the temperature dependence of a droplet’s physical properties with localized heating on the microfluidic chip, the creation of on-chip ∇*T* has been challenging as one can study microtubule polymerization^12^ along the ∇*T* or MCA for single nucleotide polymorphisms^13^, both performed in a flow-through configuration. Moreover, the MCA in microfluidics has been conducted using either solid phase multiple analysis, which requires DNA immobilization^13,14^, or the immobilization of a free liquid phase restricted for a single analysis^15^. However, micro-/nanoscale dimensions of channels and the surrounding environment and flow-through configuration may complicate the determination of the temperature distribution in thermally driven microdevices^16^. Thus, heat transfer in such a system should be characterized in heat fluxes by thermal convection, thermal radiation, or the heat transfer time between the sidewall of a chip and liquid^17,18^.

Optical systems have been used as a detector for analyzing bio/chemical samples as well as their miniaturization utilized in lab-on-a-chip (LOC) applications^19^ such as portable qPCR for DNA^20^ or RNA diagnostics^21,22^.

Recently, the combination of optics and microfluidics has attracted great attention due to the combination of highly sensitive bio-detection with LOC technology^23,24^. By fully integrating optical functions on a chip instead of using bulky external optics, these optofluidic systems lower the cost and downsize the system, making it promising for point-of-care diagnosis^25^. The detection of fluorescence remains valuable in many bio-applications. Therefore, the development and characterization of a multifunctional optofluidic lab-on-a-chip was introduced for sample analysis by fluorescence and Raman spectroscopy^26^ or by the measurement of the absorbance and fluorescence of droplets in segmented flow^27^. Further, the fluorescence detection and quantification of the Ebola virus using hybrid optofluidic integration have been reported^28^, as have platforms for real-time visualization of viruses in complex media^29^ or cell phone-based imaging cytometers^30^.

Here, we demonstrate the MCA of dsDNA with the sensitive detection of fluorescence inside a microchannel as part of an optical microsystem exposed to ∇*T* value. We used a modulated laser as the light source and a photomultiplier tube (PMT) as the fluorescence detector with its output signal processed by a lock-in amplifier to suppress environmental noise. The proposed concept of a thermally regulated optofluidic platform was first characterized in flow-through configuration of dsDNA bulk. More importantly, we presented the MCA analysis in segmented flow configuration as a tool for rapid determination of a biomolecules’ stability.

## Materials and Methods

### Chip fabrication

**Figure 1:**
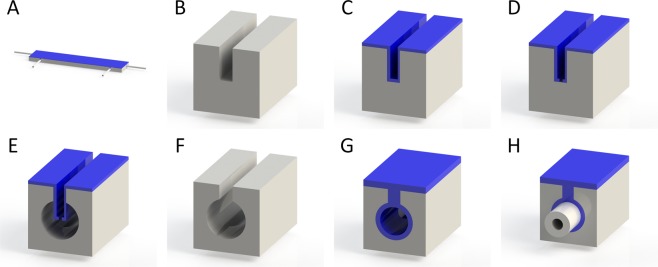
(**A**) Simplified view of whole chip with optical fibers inserted on the short edges of the chip facing each other and two capillaries inserted parallel into the long edge of the chip. (**B**) The Si substrate was patterned and etched using the Bosch process to form trenches at a width of 30 µm. (**C**) The parylene-C with thickness of ≈ 1.5 µm was deposited, and (**D**) the trench floor was later photo-blasted by femtosecond to expose the silicon. (**E**) The wafer was subjected to XeF_2_ vapors, removing silicon from the trench floor and thus forming a tubular channel with a diameter of ≈500 µm. (**F**) Parylene was stripped off using O_2_ plasma. (**G**) The wafers were diced into individual chips, and a second layer of parylene-C was deposited with a thickness of ≈ 30 µm sealing the trenches and coating sidewalls of the channels. (**H**) As the last step, the capillary and optical fibers were inserted into the side holes and sealed with an epoxy resin.

We designed the chip layout with a size of (6 × 27) mm^2^ using the Nanolithography toolbox software^31^ with the aim of having all fluid/optics inputs/outputs at the chip sidewalls to provide a robust solution. The layout consisted of ≈30 µm-wide lines subsequently forming buried microchannels by a process similar to earlier ones that used two parylene-C depositions^32^. The first parylene-C was employed as a mask with conformal coating on the sidewalls and a second parylene-C layer to seal the created channels^33^. We designed our device to insert both capillaries and the optical fibers from the sides (Fig. 1A), allowing a more robust configuration than the previous one, which had its capillaries inserted vertically.

We started the fabrication process using Si wafers with a diameter of ≈ 100 mm and an unusual thickness: ≈ 1 mm, which is thicker than the ordinary wafer thickness of ≈ 450 µm to 550 µm since the targeted microchannel diameter was the same: between ≈ 450 µm and ≈ 550 µm.

First, we coated the wafers with a ≈ 10 µm-thick positive photoresist (PR), followed by a prebake at ≈ 110 °C for ≈ 165 s. The wafers were exposed to ultraviolet light with a dose of ≈ 1600 mJ·cm^−2^ for lithography and developed in a KOH-based developer for ≈ 300 s, patterning the shape of the trenches at a designed width of 30 µm. The Si was etched with the Bosch process^34^ to a target depth of ≈ 500 µm, then, we stripped PR in N-methyl-2-pyrrolidinone solution at ≈ 80 °C for ≈ 600 s (Fig. 1B).

The wafer was then coated with a ≈ 1.5 µm parylene-C layer (Fig. 1C), and this layer was photo-blasted at the trench bottoms using a femtosecond laser working at a principal wavelength of ≈ 515 nm using pulses with a duration of ≈ 300 fs and a maximum amplitude of pulse energy of ≈ 200 μJ (Fig. 1D). The wafer was exposed to XeF_2_ vapor to isotropically etch Si through the opening in the trench bottom, forming a buried cylindrical microchannel with a diameter of ≈ 500 µm (Fig. 1E). The parylene-C was removed using O_2_ plasma with a set power of 300 W for a duration of 1 h (Fig. 1F). The wafer was diced into individual chips, and the second parylene-C layer was deposited with a thickness of ≈ 30 µm to cover the microchannel with hydrophobic materials and to seal the ≈ 30 µm-wide trench (Fig. 1G). Both optical fibers and microcapillaries were inserted into the chip and sealed with epoxy resin. Details of one port are shown in Fig. 1H.

### Optofluidic

**Figure 2:**
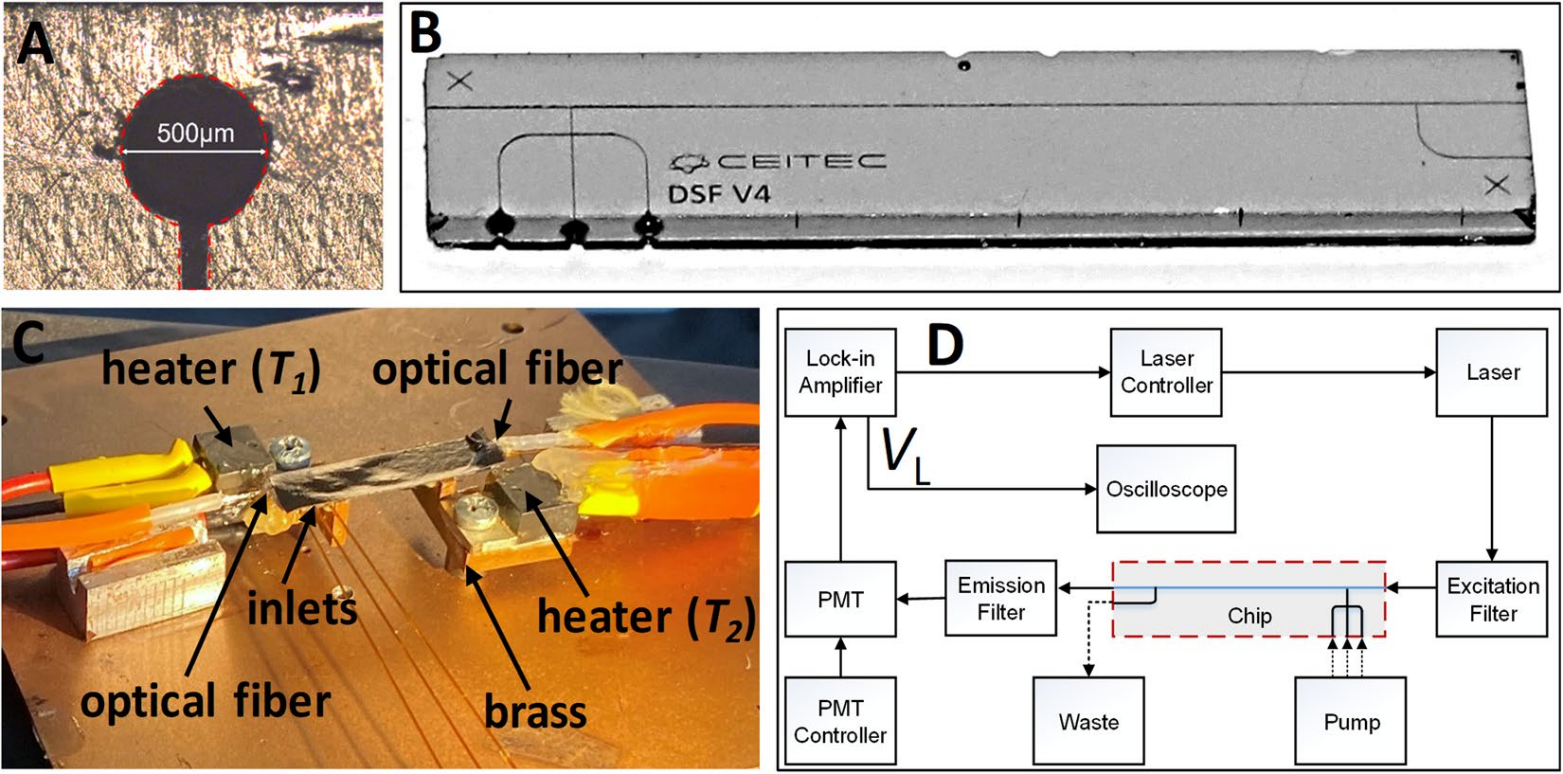
(**A**) The designed microfluidic chip had a size of ≈ (6 × 27) mm^2^. The layout consisted of two levels: the red and brown colors represent the channels and the through-holes, respectively. (**B**) Photograph of a fabricated chip made of silicon (bottom) capped. (**C**) Close-up photograph of the chip with heaters, sensors, attached optical fibers and capillaries, and (**D**) block diagram of a complete testing setup with simplified optofluidic chip layout, where *V*_L_ is the output signal from the lock-in amplifier.

The Si chip had a buried microchannel system consisting of a through channel and three inlets connected via a cross junction and a single outlet. The through channel also served as a light guide with an optofluidic path with a length of ≈ 24 mm (Fig. 2B). The diameter of the buried microchannel was chosen to be ≈ 500 µm (Fig. 2A), sufficiently large for both the optical fiber and the capillary to be inserted inside the chip to form a stable, robust, and autonomous system. The chip was placed on two brass blocks with a distance of ≈ 13 mm (Fig. 2C). Each block had its own heater and sensor connected to a proportional integrative derivative temperature controller. The heaters’ temperature was set to ≈ 50 °C and ≈ 80 °C, respectively, forming a ∇*T* value of ≈ 2.31 °C·mm^−1^. We inserted two multimode optical fibers with a core and cladding diameter of (200 ± 4) µm and (220 ± 2) µm, respectively, both (mean ± standard deviation) into the through microchannel of the chip. Each of the optical fibers was placed on an opposite side of the chip and sealed with epoxy resin. One fiber was connected to laser-producing light with a principal wavelength of 471 nm and a nominal power of 1 W. Its power was electrically modulated sinusoidal AC voltage from the lock-in amplifier’s internal power supply with a frequency and amplitude set to 1.2345 kHz and 1.958 V, respectively. The laser power entering the optofluidic chip was attenuated from its original value of 1 W by a set of neutral density filters to ≈ 25 mW. The light leaving the chip was coupled into a second optical fiber connected via a bandpass filter with a center wavelength and bandwidth of ≈ 525 nm and ≈ 50 nm, respectively, to block the second harmonic laser light with a wavelength of ≈ 471 nm and harmonic of ≈ 942 nm. We used a PMT as a light detector, having its gain set to ≈ 5 × 10^4^ by setting the control voltage to 0.67 V. The PMT output was connected to the lock-in amplifier input with sensitivity and time constant set to 50 mV per full range and 300 ms, respectively.

The high power of the laser interacting with the fluid inside the microchannel could affect the measurement as the power corresponds to the power density of ≈ 127 mW·mm^−2^. Nevertheless, the photothermal effect should not be very severe. First, the oil/water interacts with light at a rather low nominal wavelength of 471 nm, but there is, of course, an interaction with fluorescence-producing dyes, such as SYBR Green I in presence of dsDNA, which warms the water-based sample. The photothermal effect was determined earlier using 500 times more powerful laser per unit of area, which only warmed the water by ≈ 6 K^35^. Finally, we performed a relative measurement, not absolute, and thus heating of the sample by photothermal effect is compensated.

The fluid was connected to the chip via silica capillaries with internal and external diameters of ≈ 100 µm and 360 µm, respectively, to inputs and output, which were sealed with epoxy resin (Fig. 2D). We used a pressure-controlled system to control the fluid flow rate (ν). The chip was even designed to be able to generate segmented flow using a cross-junction; we formed it externally using a double T-junction as before^36^, since the externally generated segmented flow was more stable than the one formed internally.

### Chemicals

Synthetically prepared dsDNA has a length of 17 bps with a sequence of 5’-TCT GCT GTC ACA ACT AA-3’. The *T*_M_ value of the dsDNA is ≈ 60.6 °C as verified by a commercial qPCR system. We prepared the test solution by mixing this dsDNA at a concentration of ≈ 20 nM with SYBR-Green I diluted 10,000 times in a Tris-EDTA buffer (1×). We also used this buffer with no dsDNA as a reference. An oil phase of hexadecane supplemented with 2% of SPAN-80 surfactant was used as an immiscible continual phase for segmented flow analysis.

## Results and Discussion

### Mathematical calculation of heat distribution

Devices operating at elevated air temperatures with the sample flowing through are subject to heat losses by convection, radiation, and heat flux due to sample flow, possibly affecting desired temperature distribution as well as its ∇*T*^37,38^. We will only briefly describe the physical analysis, its numerical modeling, and experimental verification by infrared (IR) imaging. Details of those three methods were recently described^39^.

The system comprised a silicon chip with a thicknesses (*t*) and widths (*w*) supported by a pair of heaters (Fig. 2B), resulting in a heat flux *P*_1_ between the heaters through the microfluidic chip as:

1 $${P}_{1}=\frac{{\lambda }_{{\rm{Si}}}t\cdot w}{L}\cdot \Delta T,$$

where λ_Si_ is the thermal conductance of Si, (*L*) is the chip length, and Δ*T* is the *T* difference between the heaters. The resulting value of *P*_1_ was calculated to be ≈ 2.42 W.

The convection (*P*_2_) in air was:

2 $${P}_{2}=D\cdot w\cdot h\cdot (T-{T}_{1}),$$

where *D* is the distance between the heaters, *h* is the convection coefficient and *T*_1_ ambient temperature, giving an amplitude of *P*_2_ as ≈ 259 mW, which is 10.8% resulting in minor non-uniformity of the ∇*T* along the microchannel.

The power dissipated (*P*_3_) due to a water-based sample (sample) ν influence can be calculated by:

3 $${P}_{3}=\cdot {\rm{\upsilon }}\cdot \rho \cdot c\cdot \Delta {T}_{1},$$

where ρ is the specific mass of the sample and *c* is the sample heat capacitance.

Finally, there is also a radiation power (*P*_4_) emitted influencing the ∇*T* value. The *P*_4_ amplitude is defined by the Stefan-Boltzmann law:

4 $${P}_{4}=D\cdot w\cdot {\rm{\varepsilon }}\cdot {\rm{\sigma }}\cdot ({T}^{4}-{T}_{1}^{4}),$$

where ε is surface emissivity, and σ is the Stefan-Boltzmann constant. We calculated the values of *P*_1_, *P*_2_, *P*_3_, and *P*_4_ as before^39^ and determined the total values of (*P*_2_ + *P*_3_ + *P*_4_) are only 9% of the *P*_1_; thus, their influence on the ∇*T* can be neglected.

We neglected different temperatures along the chip to make the analytical estimation simpler. It should be done more complexly, using the integral of the function along the gradient. Nevertheless, the finite element modelling (FEM) does take the local temperature into account.

### FEM and IR imaging

**Figure 3:**
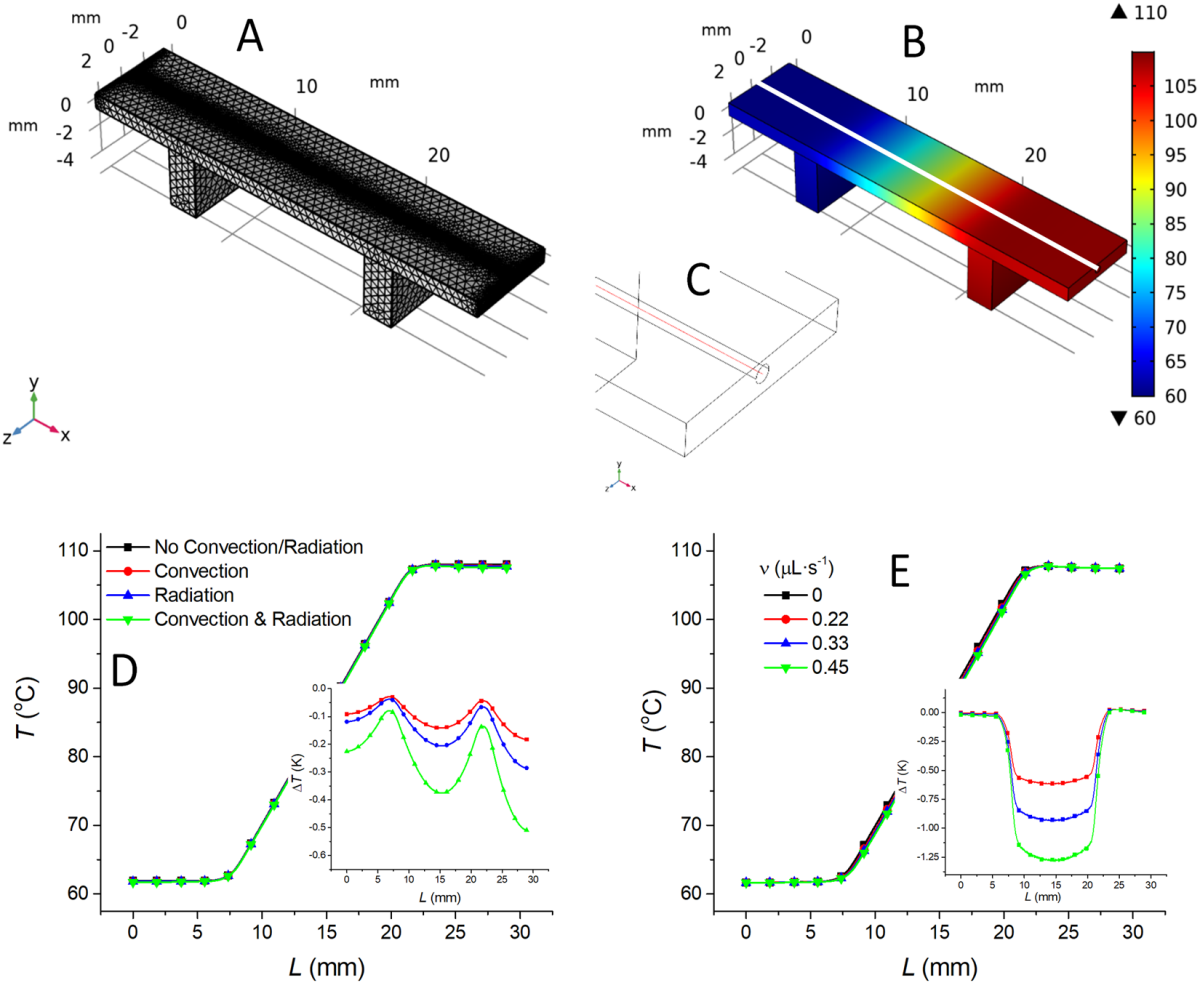
FEM of the microfluidic chip. (**A**) Mesh in the COMSOL Multiphysics. (**B**) The temperature distribution simulated by COMSOL with the heaters’ temperature value set to 50 °C and 80 °C. (**C**) A simplified sketch showing the edge of the chip with a red line in the buried microchannel center from which the temperature values were extracted. (**D**) Distribution of temperature values along the microchannel (black line) considering the influence of convection (red line), radiation (blue line), and both (green line) at zero ν. The inset is the temperature difference between models considering convection and/or radiating and the basic one using color marking corresponding to the main graph. (**E**) Temperature distribution along the microchannel with ν value as parameter. (inset) Difference between models with different ν values and the one with ν = 0 µL·s^−1^.

We modeled the microfluidic chip as well as simplified heaters in CAD software and transferred them to the finite element modeling software COMSOL Multiphysics. Then we modeled temperature distribution along the microchannel due to convection, radiation, and sample ν using *Heat Transfer in Solids and Fluids* and *Creeping Flow* modules in a fashion similar to before^39^. Here, we only show the model mesh (Fig. 3A) and calculated temperature distribution within the chip and the heaters (Fig. 3B). The simplified detail of the chip edge showing the buried microchannel is shown in Fig. 3C, where the red line shows the location of temperature data extraction for subsequent analysis. We performed FEM of the system with no external influence, with convection, radiation, and both to extract the temperature values inside the channel (Fig. 3D). We also showed the effect of convection and radiation in the inset. Finally, we checked the influence of the sample ν (Fig. 3E), shown in detail in the inset.

**Figure 4:**
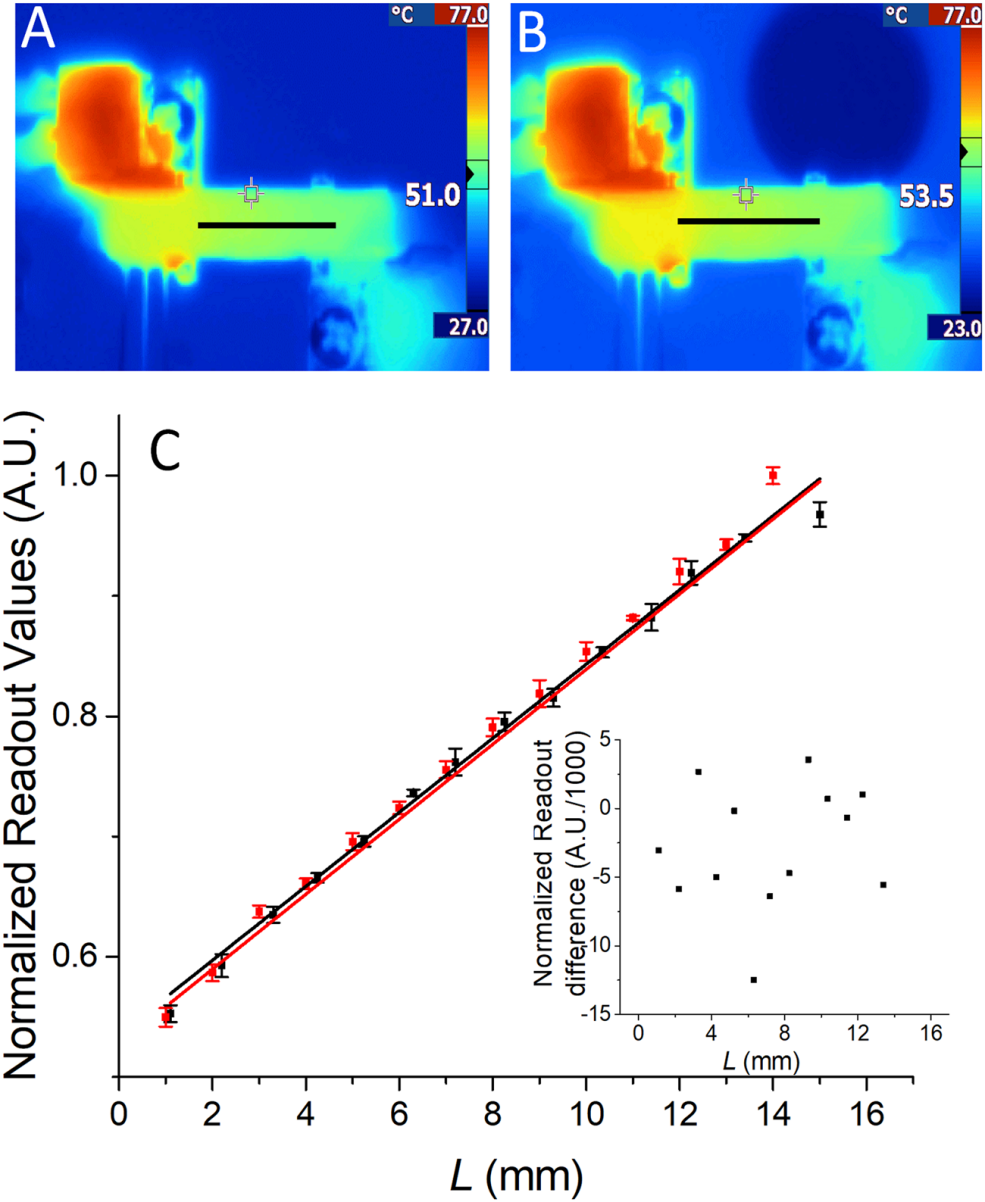
Infrared image of ∇T on a chip (**A**) without liquid flow and (**B**) with the ν of ≈ 0.5 µL·s-1. (**C**) The extracted temperature as a function of L with the slope representing the ∇T. Black line and red line represent ν of 0 µL·s-1 and ≈ 0.5 µL·s-1, respectively. Inset: the plot of difference in extracted values with and without ν.

Then we assembled the system and imaged the surface temperature using an IR camera after attaching the carbon tape on the chip surface to achieve a uniform emissivity close to unity. The chip was mounted on two heaters with the temperature set at ≈ 50 °C and ≈ 80 °C in order to create the ∇*T* along the microchannel inside the chip. The first set of IR images was captured without ν of the water sample (Fig. 4A), and the second set of images was taken with the ν set to 0.5 µL·s^−1^ (Fig. 4B), all in a monochromatic fashion. Here, we used false colors for contrast enhancement. The temperature profile alongside the center microchannel—indicated by the black line in Figs. 4A and 4B—was extracted from the IR images and plotted in Fig. 4C. We found that the value of ∇*T* between the two heaters was constant, confirming the negligible influence of convection and radiation on heat losses as simulated by FEM. The ν values of up to 0.5 µL·s^−1^ had only a marginal difference on the setup system shifting the *T* amplitude by (−2.8 ± 4.5) 0.001·A.U. (mean ± measurement error) (Fig. 4C inset). This temperature shift does show the induced influence, albeit only of a marginal value with a relatively large fitting error.

### T_M_ determination by a flow-through system

**Figure 5:**
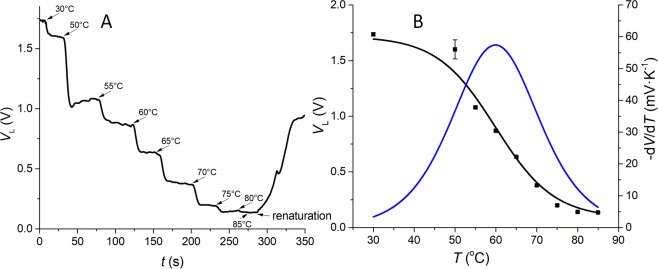
(**A**) *V*_L_ amplitude as a function of time with the temperature of both heaters set to values in the range from ≈ 30 °C to ≈ 85 °C using dsDNA with *T*_M_ value of ≈ 60.6 °C with stagnant sample. Each arrow indicates the instance when the *V*_L_ value was read for the stated temperature. (**B**) Nonlinear curve fitting of *V*_L_ values as a function of temperature extracted from (**A**) (black squares) using the Boltzmann (sigmoid) curve (black line with squares) and its derivative (blue line) to determine the value of *T*_M_. The error bars correspond to standard deviations (SD) from three measurements.

Previously, we showed a method of precise *T*_M_ determination, temperature calibration, or heat transfer rate measurement using dsDNA in the presence of a fluorescent intercalator such as SYBR Green I or Eva Green for stationary droplets^40^, as well as a flow-through system^39^. For the latter, we used a microscope to capture and analyze fluorescence images from the microchannel having ∇*T*. Here, we replaced a bulky microscope with a thermally regulated optofluidic platform to characterize the proposed detection system in a bulk sample without ∇*T*. First, we filled the microchannel with a dsDNA sample. We set *T*_1_ = *T*_2_ and gradually increased their temperature values, starting from ≈ 30 °C to ≈ 85 °C with ≈ 5 K increments while monitoring the *V*_L_ amplitude (Fig. 5A). The measurement at a determined temperature was followed by washing the solution that interacted with laser light with a fresh solution; then, we waited for ≈ 10 s for the *V*_L_ signal to stabilize the photobleaching effect. The *V*_L_ value for each temperature was read when the temperature changed on both heaters, and the signal dropped and stabilized as indicated by the arrows in Fig. 5A. Then we plotted the *V*_L_ values as a function of temperature and performed a nonlinear curve fitting using the sigmoidal Boltzmann function (Fig. 5B black line). Figure 5B also shows negative numerical derivative of *V*_L_ with respect to temperature (Fig. 5B blue line). The maximum of this curve is the value of *T*_M_ as (59.9 ± 0.2)°C (mean ± fitting error), which was close to the *T*_M_ value measured earlier using the commercial qPCR system.

### T_M_ and heat transfer determination using segmented flow

Further, we conducted an experiment using segmented flow to demonstrate a rapid MCA of the dsDNA at ∇*T*. The segmented flow was generated off-chip using a double T-junction, as it was found that the uniformity of produced segments was more stable than one generated within a heated chip.

**Figure 6:**
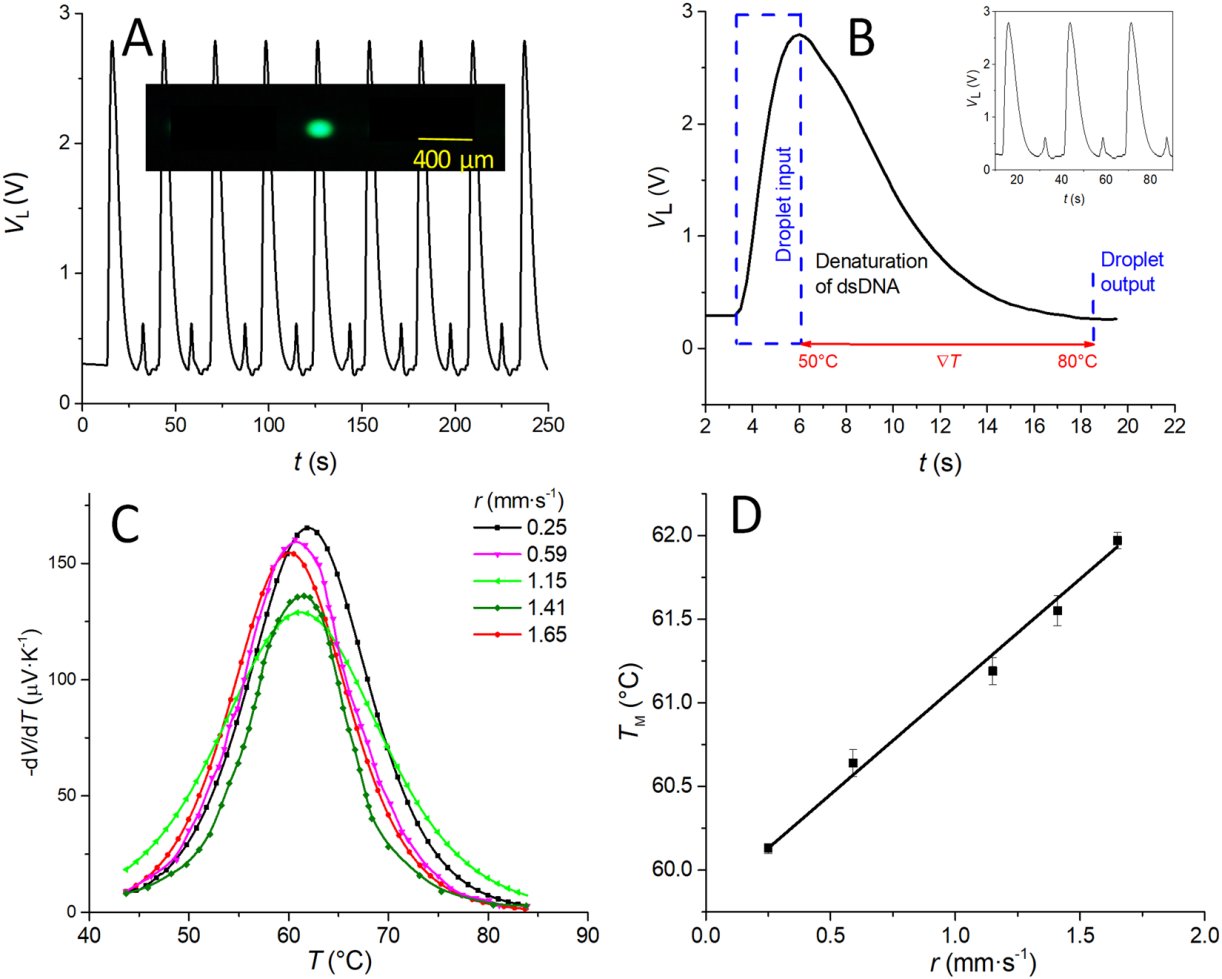
(**A**) Fluorescent emission signal from dsDNA sample and reference droplets moving across ∇*T* on the chip formed by setting the temperature of heaters at values of 50 °C and 80 °C with their distance of ≈ 13 mm. Inset shows the generated fluorescence droplet ≈ 200 µm in the microchannel. (**B**) Interpretation of fluorescence signal from a droplet passing through the ∇*T* zone in the optical microchannel with the *r* value of ≈ 1.65 mm·s^−1^. (**C**) Derivation of fluorescence amplitude generated from a droplet as a function of *r*. (**D**) Plot of *T*_M_ values as a function of *r*.

We controlled the droplet generation as well as the ratio between oil and water phases using external *p* values set from ≈ 25 kPa to ≈ 75 kPa, respectively. We recorded the *V*_L_ signal for ≈ 250 s, getting its value to alternate between ≈ 300 mV and ≈ 3 V for reference droplets and the sample, respectively (Fig. 6A), with the length of the droplet estimated to be ≈ 200 µm (Fig. 6A inset).

The *V*_L_ amplitude increases above the baseline corresponding to the droplet entering the optofluidic path, reaching the maximum when the whole droplet is inside the optofluidic microchannel (Fig. 6B). The droplet there was exposed to ∇*T* inside the microchannel; thus, the value of *V*_L_ dropped. This decrease of the *V*_L_ value is nonlinear due to the nature of dsDNA’s melting characteristic. We extracted the part of the curve corresponding to ∇*T* exposure (red arrowed line) and performed a non-linear curve fitting using the Boltzmann (sigmoid) function with respect to time, subsequently converting time to location and temperature^39^. The fitted curve was processed by derivation -d*V*/d*T* (Fig. 6C) with flow speed (*r*) values (Table 1) as a parameter. The values of *r* were determined by the time (*t*) required for a droplet to pass the whole length of ≈ 24 mm of microfluidic microchannel and then *r* = *t*/24. Subsequently, we expressed the *T*_M_ as a function of *r* values (Table 1 and Fig. 6D). Figure 6D shows how the *T*_M_ values increase with increasing *r* corresponding to the heat transfer rate. From this curve, the slope can be extracted to calculate the time required for heat to reach the center of the microchannel from the channel sidewalls.

**Table 1:** Melting temperature as function of flow rates in droplet-based configuration, showing the duration for MCA as the value of time (*t*) required for the droplet to pass though the microchannel.

Set *p* (kPa)	*ν* (µL·s^−1^)	*t* (s)	*r* (mm·s^−1^)	*T*_M_ (°C) (mean ± SD)
≈25	0.22	≈96.0	≈0.25	60.13 ± 0.03
≈40	0.28	≈40.2	≈0.59	60.64 ± 0.08
≈50	0.33	≈20.9	≈1.15	61.19 ± 0.08
≈60	0.37	≈17.0	≈1.41	61.55 ± 0.04
≈75	0.45	≈14.5	≈1.65	61.97 ± 0.05

Thus, we performed linear fitting to obtain the slope (*S*) as (1.28 ± 0.04) K·s·mm^−1^ (mean ± fitting error). The *S* value divided by ∇*T* of ≈ 2.31 K mm^−1^ produces a heat transfer time of ≈ 554 ms. Knowledge of this value can be used to correct the system results when they are collected at different values of *r*.

The MCAs are typically performed using commercial real-time PCR systems with a typical temperature ramp rate between ≈ 0.1 and ≈ 0.5 K·s^−1^, thus corresponding to 500 s to 100 s per test (50 K temperature ramping), excluding sample loading^41^. An ultrafast method to perform the MCA in an astonishing ≈ 50 ms has been reported^42^. It was based on a droplet placed on a cold Cu substrate with the sample heated by laser irradiation that required a complex setup and careful sample preparation. Our optofluidic platform enables rapid MCA of the sample containing biomolecules such as DNA or proteins. The time needed for the analysis is less than ≈ 30 s, which makes this platform faster than other systems. That can significantly speed up the MCA and make it suitable for high-throughput screening.

## Conclusion

We proposed an optofluidic chip having ∇*T* along the microchannel to perform the MCA of dsDNA or protein unfolding. We first numerically analyzed the influence of convection, radiation, and a sample ν on the temperature distribution, as well as ∇*T* deviation from a constant value. The optofluidic chip was equipped with a buried microchannel with a diameter of ≈ 500 µm. We inserted both optical fibers and microcapillaries inside the chip, forming an integrated and robust autonomous system. The microfluidic channel served as a light guide for sensitive fluorescence detection along the ∇*T* inside the chip. We demonstrated this concept by determining the dsDNA *T*_M_ in a continuous-flow configuration. We also conducted the MCA in a droplet-based configuration using segmented flow. This concept of fluorescence monitoring in a microchannel exposed to ∇*T* represents a fast and cost-effective approach for the characterization of thermal properties of biomolecules. It could be crucial in many areas of biology and chemistry, including the stability of proteins, by exposing them to different environments such as pH, buffer composition and ionic strength, purity control, and protein-ligand interaction. Those applications could have a great impact on drug discoveries as well as the molecular diagnostics of infectious diseases or genotyping.
